# Wear Behavior of Laser-Cladded TiN-Reinforced AlCoCrFeNi High-Entropy Alloy Coatings on 304 Stainless Steel

**DOI:** 10.3390/ma19122563

**Published:** 2026-06-13

**Authors:** Qian Deng, Ying Wang, Yuxuan Liu, Zhigang Hu, Ming Ma, Mao Zhang, Yong Ai

**Affiliations:** 1 College of Artificial Intelligence, Wuchang University of Technology, Wuhan 430223, China; 2 School of Mechanical Engineering, Wuhan Polytechnic University, Wuhan 430023, China; 3 State Key Laboratory of Materials Processing and Die & Mould Technology, School of Materials Science and Engineering, Huazhong University of Science and Technology, 1037 Luoyu Road, Wuhan 430074, China; 4 Hubei Lioho-Tianlun Machinery Co., Ltd., No. 1 Chuangye Road, Xiantao Industry Zone, Xiantao 433001, China

**Keywords:** laser cladding, high-entropy alloys, friction and wear, microstructure

## Abstract

*AlCoCrFeNi* high-entropy alloy coatings reinforced with different TiN contents (2 wt.%, 4 wt.%, and 6 wt.%) were fabricated on 304 stainless steel by laser cladding. The effects of TiN addition on the microstructure, hardness, friction behavior, and wear resistance of the coatings were investigated. Dry reciprocating sliding tests were conducted under a load of 10 N, a frequency of 5 Hz, a stroke length of 5 mm, and a duration of 20 min using GCr15 bearing steel balls as the counterpart. The results showed that the 2 wt.% TiN coating exhibited the best tribological performance within the investigated composition range, with a microhardness of 579.6 HV_0.5_, a relatively low and stable friction coefficient of approximately 0.30–0.35, and a wear rate of 2.9 × 10^−4^ mm^3^/(N·m). When the TiN content increased to 4 wt.% and 6 wt.%, the wear resistance decreased, which was mainly associated with particle agglomeration, local stress concentration, and brittle spalling. These results indicate that appropriate TiN addition can improve the load-bearing capacity and wear resistance of laser-cladded *AlCoCrFeNi* coatings, providing a potential surface-strengthening strategy for 304 stainless steel components under dry sliding conditions.

## 1. Introduction

In the modern high-end equipment manufacturing field, 304 stainless steel is often used as the basic material for core moving parts and load-bearing components (such as locating pins, spline gears, etc.) due to its low cost, good processing performance, and stable comprehensive mechanical properties [1]. However, as mechanical equipment gradually develops towards higher speeds, higher loads, and longer service life, its key components often need to withstand complex working conditions such as continuous friction, impact loads, and abrasive cutting during service. For 304 stainless steel, its surface hardness and resistance to plastic deformation are limited [2,3,4], and it is prone to failure phenomena such as spalling and fatigue wear under heavy-load sliding friction or particle wear environments, resulting in decreased surface accuracy, increased material loss, and shortened service life of the components. Therefore, improving the wear resistance of 304 stainless steel through surface modification techniques [5] is crucial for ensuring its long service life and high reliability under harsh conditions [6,7]. Traditional surface modification methods include thermal spraying, nitriding, carburizing, electroplating, and chemical plating [8,9,10]. These methods can improve the surface properties of the material to a certain extent, but they still have problems such as high porosity of the coating, insufficient bonding strength, and limited thickness [11,12]. In contrast, laser cladding technology uses a high-energy laser beam to rapidly melt and solidify the alloy powder and the surface layer of the substrate, forming a dense, low dilution rate, and metallurgically bonded coating on the substrate surface [13,14]. This technology has advantages such as concentrated heat input, small heat affected zone, flexible composition design, and higher automation [15], and is suitable for strengthening and wear-resistant repair of the surfaces of large mechanical components.

In recent years, high-entropy alloys have attracted significant attention in the field of laser cladding coatings due to their multi-component composition and excellent microstructure control capabilities [16,17]. Unlike traditional alloys, high-entropy alloys are generally composed of five or more elements in an equal atomic ratio [18], and form unique microstructures and strengthening characteristics under the combined effect of multiple components. The lattice distortion effect [19], sluggish diffusion effect [20], and solid solution strengthening effect [21,22] enable high-entropy alloy coatings to demonstrate significant application potential in terms of hardness, strength, and wear resistance, making them an important research direction for metal surface strengthening [23,24]. Among various systems, *AlCoCrFeNi* has excellent laser cladding forming adaptability and microstructure control space, which can improve the bearing and anti-wear performance of the coating to a certain extent [25]. However, when facing harsh conditions such as long-term sliding friction, high contact stress, or abrasive cutting, a single *AlCoCrFeNi* coating is still prone to local plastic deformation [26], surface peeling, and increased wear.

To further enhance the service stability of the coating in complex friction environments, the introduction of hard ceramic reinforcing phases has become an effective way to improve the wear resistance of high-entropy alloy coatings [27]. Ceramic particles such as TiC, WC, SiC, and TiN usually have high hardness, high melting point, and good thermal stability [28] and can improve the wear resistance of the coating through mechanisms such as dispersion strengthening and fine-grain strengthening. Among them, TiN is considered a potential wear-resistant reinforcing phase due to its high hardness and good thermal stability [29]. Previous studies have reported that TiN or ceramic-particle reinforcement can effectively improve the hardness and wear resistance of high-entropy alloy coatings. Liu et al. [30] prepared in situ TiN-reinforced FeCoNiCrMnTi high-entropy alloy coatings on 304 stainless steel by laser cladding and found that TiN formation contributed to improved hardness and wear resistance. Wang et al. [31] also fabricated in situ TiN-reinforced FeCoNiCrMnTi coatings and reported that TiN addition enhanced the coating hardness and improved resistance to adhesive and oxidative wear. In addition to high-entropy alloy coatings, Manokhin et al. [32] demonstrated that TiN-coated cBN powders could reduce stress concentration and improve wear resistance during high-speed cutting, indicating the positive role of TiN in load-bearing and wear-resistant systems. Liang et al. [33] investigated in situ TiN-reinforced AlCoCrCuNiTi high-entropy alloy coatings and revealed that the interfacial bonding between TiN and the matrix played an important role in improving wear resistance.

The aforementioned research indicates that TiN has demonstrated significant advantages in the wear resistance of the high-entropy alloy coating. However, the mechanism by which different TiN addition amounts affect the wear resistance of the *AlCoCrFeNi* laser cladding coating remains unclear. The synergistic strengthening effect between TiN particles and the multi-component matrix, as well as the quantitative correlation between the microstructure evolution of the coating and the macroscopic friction and wear performance, have not been systematically studied.

Previous studies have mainly focused on TiN-reinforced high-entropy alloy coatings with different alloy systems or in situ ceramic formation, while the content-dependent effect of TiN addition on the microstructure evolution and wear response of laser-cladded *AlCoCrFeNi* coatings has not been sufficiently clarified. In particular, the relationship among TiN content, BCC phase evolution, hard-particle distribution, friction-coefficient stability, wear-scar geometry, and worn-surface damage features remains unclear. Therefore, this work focuses on *AlCoCrFeNi* coatings with different TiN additions and systematically correlates the microstructural evolution with hardness, friction behavior, wear rate, and wear mechanism. The main contribution of this study is to reveal the threshold effect of TiN addition, showing that excessive TiN does not continuously improve wear resistance but instead promotes particle agglomeration, brittle spalling, and unstable friction behavior.

## 2. Experimental Materials and Methods

### 2.1. Coating Materials

In this experiment, spherical *AlCoCrFeNi* high-entropy alloy powder (with a purity of ≥98% and a particle size of 40–115 μm) prepared by vacuum atomization and TiN ceramic powder (with a purity of ≥99.9% and a particle size of 1–3 μm) were used as raw materials. TiN powder was added to the *AlCoCrFeNi* alloy powder at mass fractions of 2 wt.%, 4 wt.%, and 6 wt.%, and the corresponding samples were labeled as A, B, and C, respectively. These TiN contents were selected to represent low, medium, and relatively high ceramic-particle additions so as to evaluate the content-dependent effect of TiN on the microstructure and wear behavior of the *AlCoCrFeNi* coating. The powder mixtures were placed in sealed plastic bottles together with 304 stainless steel balls for ball-assisted rolling mixing. The ball-to-powder mass ratio was 3:1, and the bottles were continuously rolled in a mixing machine at a rotational speed of 300 r/min for 48 h under ambient air atmosphere. The use of 304 stainless steel balls and plastic bottles was intended to promote uniform powder mixing while reducing severe powder crushing during the mixing process. Before mixing, the plastic bottles and 304 stainless steel balls were cleaned with absolute ethanol and dried. After mixing, the composite powders were dried in a vacuum drying oven (DZF-6090, Bluepard, Shanghai, China) at 150 °C for 2 h and then stored in sealed bags for subsequent laser cladding.

### 2.2. Optimization of Laser Cladding Process Parameters

**Figure 1:**
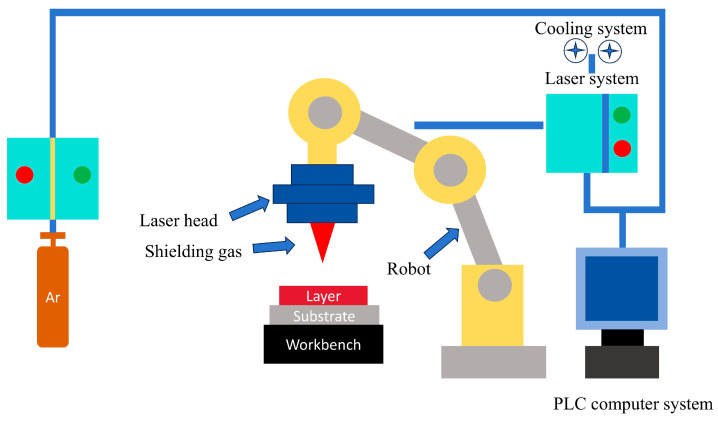
Schematic diagram of the laser cladding system.

The laser cladding processing system used in this study was a LASERTEC 65 system (DMG MORI, Bielefeld, Germany), mainly consisting of a laser generation system, a cooling system (DL20-900, Dongluyang, Shenzhen, China), a Siemens SIMATIC S7-1500 PLC (SIEMENS Aktiengesellschaft, Munich, Germany) control system, and an ABB IRB 6700 robotic arm (TKB3670, TURINSOFT, Shanghai, China). The schematic diagram of the laser cladding system is shown in Figure 1. The 304 stainless steel substrates were machined into dimensions of 100 mm × 100 mm × 6 mm. Before laser cladding, the substrate surfaces were ground with SiC papers, cleaned with absolute ethanol, and dried. Before use, the mixed powders with different TiN contents were dried at 150 °C for 2 h to remove moisture. A single-layer multi-track coating was prepared on the substrate surface by laser cladding. The coating length and width were approximately 20 mm and 20 mm, respectively, and the coating thickness was approximately 1.5–2.0 mm. The laser spot diameter was 4 mm, and the overlap ratio between adjacent cladding tracks was 50%, corresponding to a hatch spacing of 2 mm. No preheating treatment was applied to the substrate before laser cladding. Argon was used as the shielding gas with a flow rate of 15 L/min. The main laser cladding parameters, including laser power, scanning speed, powder feeding rate, shielding gas flow rate and spot diameter, are listed in Table 1. These parameters were determined based on preliminary process optimization, with coating forming quality, metallurgical bonding, and defect control considered as the main evaluation criteria. To prevent cross-contamination among different powder compositions, the powder-feeding pipeline was thoroughly cleaned with compressed air, and the powder container was cleaned before preparing each coating.

### 2.3. Characterization Methods

The phase composition of the coatings was analyzed by X-ray diffraction (XRD, X’Pert3 Powder, PANalytical B.V., Almelo, The Netherlands) using Cu Kα radiation (λ = 0.15406 nm). The scanning range was 10–80°, with a scanning rate of 8°/min. Phase identification was performed using the PDF database.

The powder morphology, coating microstructure, cross-sectional element distribution, and worn surfaces were characterized by environmental scanning electron microscopy (ESEM, FEI, Hillsboro, OH, USA) equipped with an energy-dispersive spectroscopy system. The accelerating voltage was 20 kV. EDS mapping and point analysis were performed on representative regions of the powders, coating cross-sections, and worn surfaces. The EDS results were used mainly for semi-quantitative compositional comparison.

The three-dimensional morphologies and cross-sectional profiles of the wear tracks were measured using a NewView 8200 white-light interferometer (Zygo Corporation, Middlefield, CT, USA).

### 2.4. Microhardness and Friction and Wear Testing

The microhardness of the samples was measured using a fully automatic Vickers hardness tester (Huayin, Laizhou, China) (test load 4.9 N, holding pressure for 10 s). Five points at an interval of 150 μm were selected for testing in each group. All friction and wear tests were conducted under dry sliding conditions in ambient air at room temperature, without the use of any lubricant. The results were presented as “average value ± standard deviation”. At room temperature, the friction and wear test was conducted using a (MS-M9000 type) linear reciprocating testing machine (Huahui, Lanzhou, China). The counterpart balls were GCr15 bearing steel balls with a diameter of 4 mm and a mirror-polished surface. GCr15 bearing steel is equivalent to AISI 52100 bearing steel, and the nominal hardness of commercial GCr15 steel balls is generally HRC 62–66. Before the wear tests, the coating surfaces were ground and polished to a mirror-like finish to ensure comparable initial surface conditions. The experimental parameters were set as a normal force of 10 N, frequency of 5 Hz, reciprocating length (amplitude) of 5 mm, and duration of 20 min. Before the experiment, the samples and the counterpart balls were cleaned and dried. A new counterpart ball was used for each wear test to avoid the influence of previous wear damage on subsequent measurements. Each test was repeated 3 times and the friction coefficient (coefficient of friction) curve was automatically recorded. The instantaneous friction coefficient is calculated as μ = Ft/Fn, where *Ft* is the tangential friction force recorded by the tribometer, and *Fn* is the applied normal load. Considering the fluctuations during the initial running-in stage, the average friction coefficient is calculated from the steady-state region of the friction curve. After the wear tests, the three-dimensional morphology of the wear tracks was obtained using a NewView 8200 white-light interferometer (Zygo Corporation, Middlefield, CT, USA), and the cross-sectional profiles were extracted for wear-volume calculation. Before extracting the wear depth and cross-sectional profiles, the 3D wear-track data were leveled using the unworn regions on both sides of the wear track as the reference plane, and the average height of the original unworn surface was set as zero. Therefore, the negative displacement below this reference plane represents material loss, whereas the positive displacement near the wear-track edges mainly corresponds to edge pile-up. Since the wear tracks showed relatively regular morphologies, the wear volume was calculated by the cross-sectional profile method. For each measured 3D wear-track area, three cross-sectional profiles perpendicular to the sliding direction were extracted from different representative positions along the wear track, excluding the two turning ends of the reciprocating path. The wear cross-sectional area of each profile was determined by integrating the material loss below the zero reference line using Origin software (2024b). The average cross-sectional area of the three profiles was then multiplied by the effective wear-track length to obtain the wear volume. The coating wear rate was calculated using the formula W = V/(F × L), where *V* is the wear volume (mm^3^), *F* is the normal load (N), and *L* is the total sliding distance (m). The uncertainty of the wear-rate measurement was expressed as the standard deviation of three repeated tests.

## 3. Results and Discussion

### 3.1. Morphology, Phase Composition, and Elemental Distribution of Laser-Cladded Powders and Coatings

**Figure 2:**
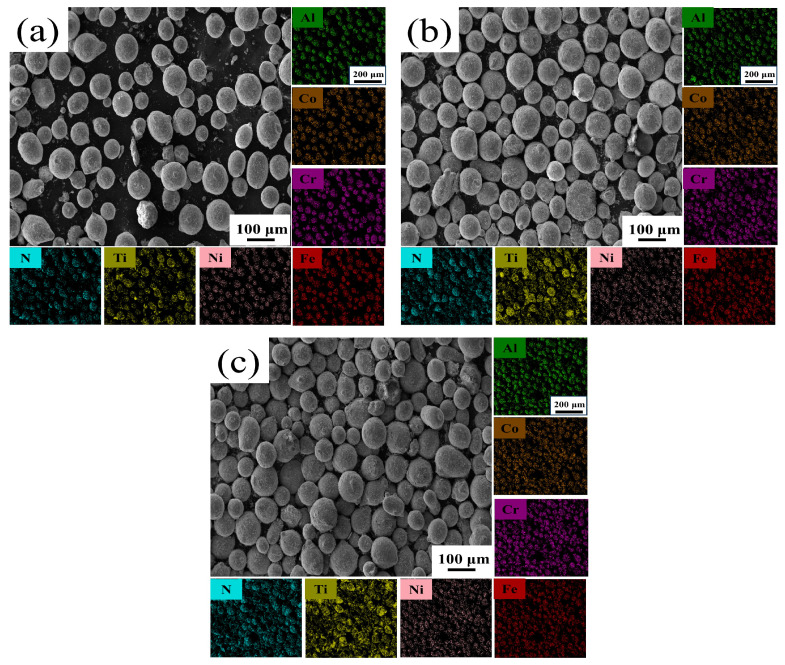
SEM morphology and corresponding EDS elemental maps collected from the same observation fields of laser cladding powder mixtures: (**a**) sample A (2 wt.% TiN); (**b**) sample B (4 wt.% TiN); (**c**) sample C (6 wt.% TiN).

The SEM morphology analysis is shown in Figure 2 The high-entropy alloy powders of samples A, B, and C all exhibit standard spherical characteristics and have uniform particle size distribution. The EDS elemental maps in Figure 2 were collected from the same SEM observation fields as the corresponding powder morphology images so as to directly correlate the powder morphology with the elemental distribution. This excellent sphericality enables the powders to have excellent fluidity, effectively preventing blockage during the powder feeding process, thereby ensuring the stability of the high-entropy alloy coating. Further, SEM-EDS analysis showed that the main elements of Al, Co, Cr, Fe, Ni, Ti, and N were detected in the samples, indicating that the TiN-containing mixed powders/coatings were compositionally distinguishable among the different groups. The chemical compositions listed in Table 2 were obtained from SEM-EDS area analysis of selected regions. Therefore, these values should be regarded as semi-quantitative results and mainly used to compare the element distribution tendency among different samples rather than to represent the exact bulk composition of the mixed powders. In particular, the quantification of light elements such as N by EDS may involve relatively large uncertainty.

**Figure 3:**
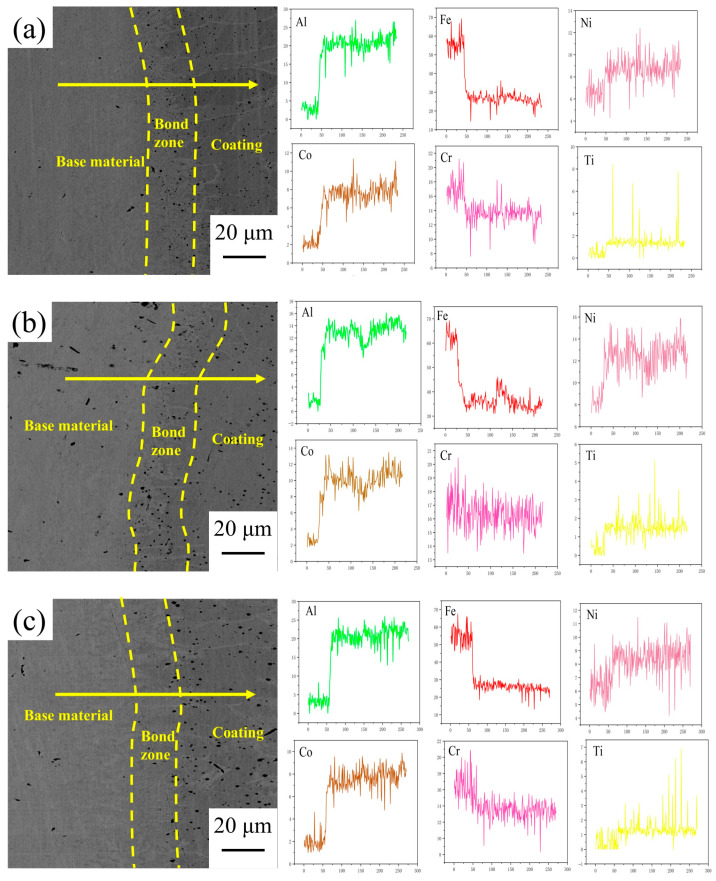
Cross-sectional line scanning images of the HEA cladding layer/stainless steel substrate: (**a**) sample A; (**b**) sample B; (**c**) sample C.

The cross-sectional EDS line scanning characterization of samples A, B, and C was conducted (Figure 3), revealing that a transition bonding zone with a thickness of approximately 10–15 μm was formed at the interface of all samples. From the fluctuation trend of the scanning curves, the Fe element showed a significant concentration change and diffused into the coating, while other elements such as Al, Ti, Ni, and Cr also entered the stainless steel matrix in small amounts. This suggests that a tight metallurgical bond was formed between the coating and the substrate.

**Figure 4:**
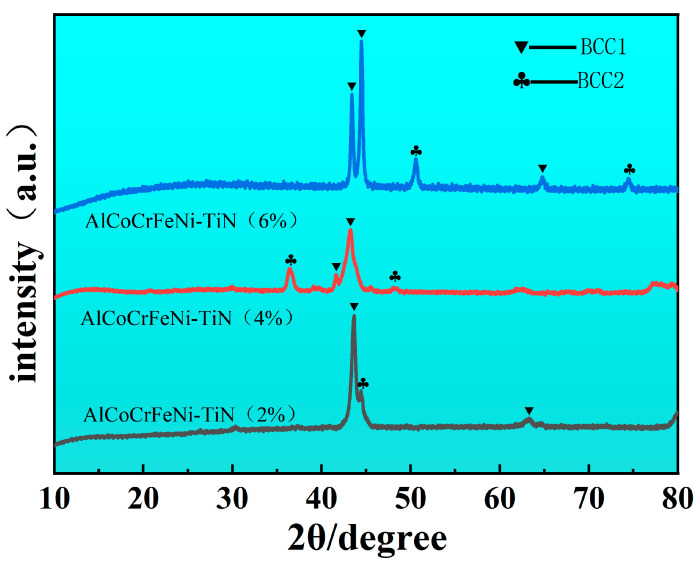
XRD patterns of the coatings of samples A, B and C.

Figure 4 shows the XRD patterns of the coatings of samples A, B, and C. From the figure, it can be seen that the coating matrix is mainly composed of the typical body-centered cubic solid solution (BCC1 phase). In addition, the body-centered cubic second phase (BCC2 phase) was observed in the coating. As the content of TiN in the *AlCoCrFeNi* high-entropy alloy increases, the intensity of the diffraction peak of BCC2 phase significantly enhances. The precipitation of this BCC2 phase is due to the introduction of TiN particles. The addition of TiN intensifies the phase separation and lattice distortion within the *AlCoCrFeNi* high-entropy alloy matrix, thereby promoting the formation of the BCC2 s phase in the coating.

**Figure 5:**
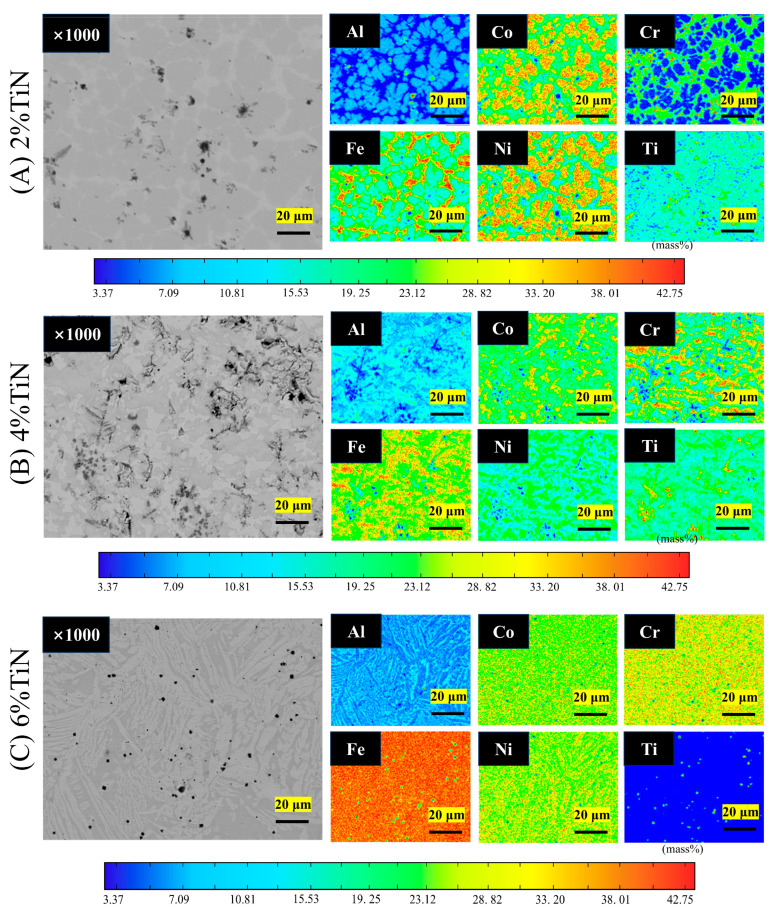
Initial morphology and elemental composition distribution of AlCoCrFeNi high-entropy alloy coatings doped with 2 wt.%, 4 wt.%, and 6 wt.% TiN.

Figure 5 shows the microstructure morphology and element distribution characteristics of the *AlCoCrFeNi* high-entropy alloy coating under three different TiN contents (2 wt.%, 4 wt.%, and 6 wt.%) at a 1000-fold magnification.

From the SEM micrographs (A, B, and C) and the corresponding elemental distribution maps in the figure, it can be observed that all three coatings exhibit a distinct bimodal structure. When the TiN content is 2 wt.% and 4 wt.%, (Figure 5A,B), the coatings are mainly composed of typical dendritic crystals and the inter-dendritic structure. Combining the elemental distribution, it can be seen that the Al and Ni elements tend to be enriched in the same area, while the Fe, Co, and Cr elements are mainly enriched in another area (i.e., the solid solution phase), showing a significant component segregation. As the TiN addition amount increases, this phase separation phenomenon becomes more pronounced; especially when the TiN content reaches 6 wt.%, (Figure 5C), the microstructure of the coating undergoes a significant transformation, changing from dendritic crystals to a regular and dense alternating layer-platelet/maze-like structure, indicating that the high content of TiN promotes the formation of BCC and other Al/Ni-rich phases and the transformation of the phase structure.

The Ti elemental maps further reveal the distribution behavior of Ti-containing regions in the coatings. For the 2 wt.% and 4 wt.% TiN coatings, the Ti-related signals were relatively dispersed in the matrix, indicating that the Ti-containing reinforcing phase was more uniformly distributed. With further increasing the TiN content to 6 wt.%, obvious Ti-rich regions appeared in the elemental map, suggesting local aggregation of Ti-containing particles. This aggregation may weaken the structural continuity of the *AlCoCrFeNi* matrix and act as potential sites for stress concentration during sliding wear. It should be noted that the SEM-EDS results are mainly used to reveal the element distribution tendency, while the accurate identification of possible nanoscale nitride phases requires further high-resolution characterization. Therefore, no additional nitride phase is assigned here beyond the phases supported by the XRD results.

### 3.2. Friction Behavior and Wear Mechanism of AlCoCrFeNi High-Entropy Alloy Coatings Doped with TiN

**Figure 6:**
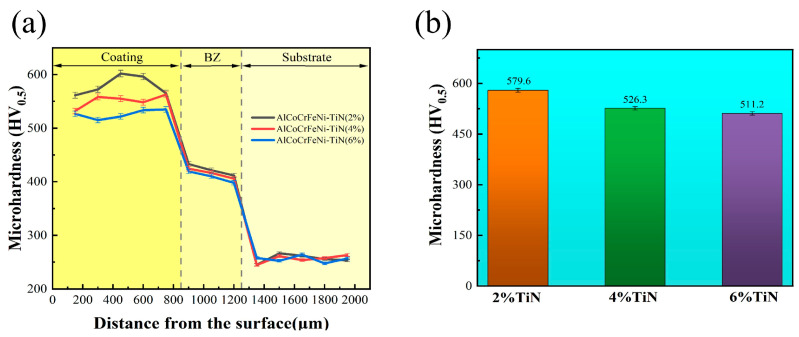
(**a**) Microhardness distribution of the coating cross-section along the depth direction; (**b**) average microhardness of the coatings of samples A, B, and C.

The results of the microhardness test along the depth direction are shown in Figure 6a. The hardness curve is clearly divided into three distinct hardness regions: the coating zone, the bonding zone, and the substrate zone. Within the coating, the standard deviation of hardness at each measurement point is extremely small, which proves the uniformity of the coating composition and the stability of the microstructure [34].

The coating of sample A achieved its peak average hardness (579.6 HV_0.5_) due to the uniform dispersion strengthening effect; the hardness of samples B and C showed a downward trend (the hardness of sample C coating dropped to 511.2 HV_0.5_). This decrease in hardness is caused by the excessive hard phases aggregating during solidification, which not only weakens the dispersion strengthening effect [35,36] but also easily leads to local stress concentration.

**Figure 7:**
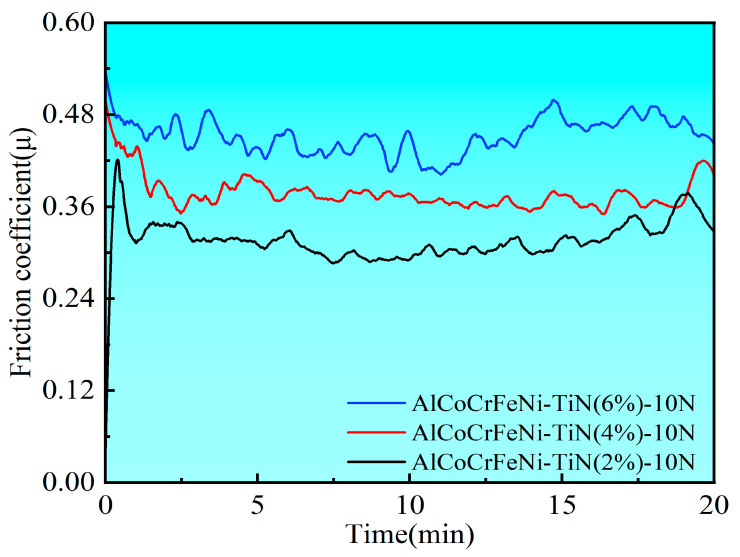
Friction coefficients of coatings on samples A, B, and C under a 10 N load.

Figure 7 shows the representative friction-coefficient curves of samples A, B, and C under a 10 N load. The average coefficient of friction was calculated from the steady-state region after excluding the initial running-in stage. Each test was repeated three times, and the standard deviation of the repeated tests was used to evaluate the repeatability and fluctuation of the friction behavior. Sample A exhibited the lowest and most stable friction coefficient, whereas samples B and C showed higher friction coefficients and more obvious fluctuations. The analysis suggests that the appropriate dispersion of hard ceramic phases within the matrix effectively enhanced the load-bearing capacity of the coating surface [37]. During dry sliding, this relatively stable surface state was conducive to the formation of an oxide-containing mechanically mixed layer on the worn surface, which reduced direct contact between the counterpart ball and the coating and helped stabilize the friction response [38].

Under a 10 N load, the curves of the coatings of samples B and C both exhibited significant fluctuations and higher means. Among them, the friction coefficient of the coating group of sample C was the highest, accompanied by intense sawtooth-like oscillations (oscillating between 0.40 and 0.50). This unstable friction state is usually associated with high roughness wear at the interface and local fatigue spalling. Combined with the microstructure analysis, excessive TiN particles are prone to agglomeration, resulting in increased coating brittleness and easy triggering of fatigue failure under local stress concentration; at the same time, under the action of dry sliding wear shear force, these hard agglomerates are prone to discontinuous peeling and form a large amount of debris. These hard abrasive particles formed a “three-body wear” mode between the mating parts and the coating, causing the friction pair to transition from “sliding friction” to “grinding state”, frequently disrupting the mechanical balance of the friction interface and resulting in significant fluctuations in friction resistance.

In conclusion, the addition amount of TiN has a significant window effect on the improvement in the frictional properties. An excessively high addition amount will cause material brittleness and the shedding of hard particles, thereby disrupting the dynamic stability of the interface and resulting in poor wear resistance. The experimental results indicate that the 2 wt.% TiN coating achieved a better balance between hard-phase strengthening and microstructural continuity, which contributed to its lower and more stable friction coefficient and improved wear resistance.

**Figure 8:**
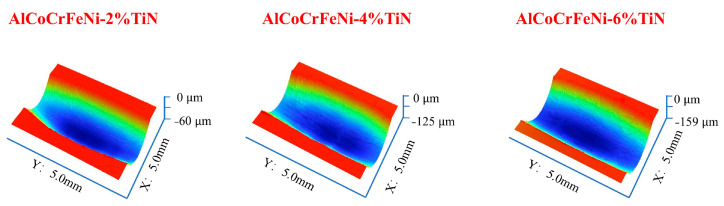
Three-dimensional surface morphology images of samples A, B, and C after 10 N friction test.

The three-dimensional wear morphologies were obtained using a white-light interferometer, as shown in Figure 8. To ensure consistency between the three-dimensional morphologies and the cross-sectional profiles, the 3D data were reprocessed using the unworn regions on both sides of the wear track as the reference plane. The average height of the original unworn surface was set as zero, and the maximum wear depth was defined as the vertical distance from this reference plane to the lowest point inside the wear track. Under a 10 N load, the maximum wear depths of the 2 wt.%, 4 wt.%, and 6 wt.% TiN coatings were 60 μm, 125 μm, and 159 μm, respectively.

The 2 wt.% TiN coating exhibited the smallest wear depth and a relatively smooth wear-track bottom, indicating that an appropriate amount of TiN improved the load-bearing capacity and resistance to plastic deformation of the coating. In contrast, the 4 wt.% and 6 wt.% TiN coatings showed significantly increased wear depths, more obvious grooves, and irregular peeling pits. This deterioration was mainly associated with the local agglomeration of excessive TiN-containing hard phases, which weakened the structural continuity of the coating and promoted stress concentration during reciprocating sliding. Under repeated shear stress, cracks could initiate near these agglomerated regions and propagate toward the worn surface, resulting in local brittle spalling and rougher wear-track morphologies.

**Figure 9:**
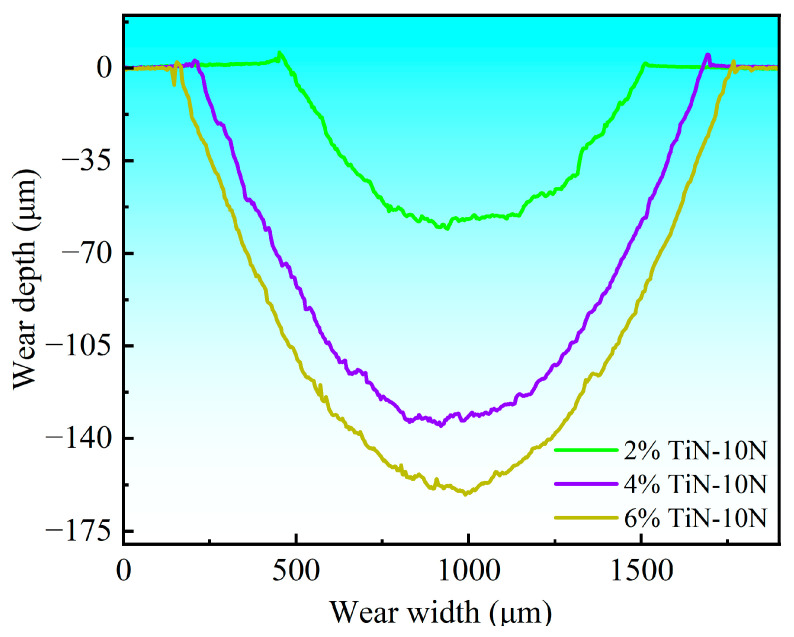
Cross-sectional profiles of the wear marks on the coatings of samples A, B, and C after the friction and wear test.

As shown in Figure 9, the cross-sectional profiles were extracted from the reprocessed three-dimensional wear-track data using the same unworn-surface reference plane as that used in Figure 8. The zero line represents the average height of the original unworn surface. Therefore, the negative displacement below the zero line corresponds to material loss, while the positive displacement near the wear-track edges mainly reflects edge pile-up.

Under a 10 N load, the cross-sectional profiles became wider and showed greater material loss with increasing TiN content. The maximum wear depths determined from the same reference plane were 60 μm, 125 μm, and 159 μm for the 2 wt.%, 4 wt.%, and 6 wt.% TiN coatings, respectively. Compared with the 2 wt.% TiN coating, the 4 wt.% and 6 wt.% TiN coatings exhibited larger profile fluctuations, deeper grooves, and more obvious local peeling pits, which were consistent with the three-dimensional wear morphologies shown in Figure 8.

The increased wear depth and profile irregularity of the coatings with higher TiN contents can be attributed to local failure caused by stress concentration. For the 4 wt.% and 6 wt.% TiN coatings, the cross-sectional profiles showed more obvious zigzag fluctuations at the bottom of the wear tracks. This behavior was mainly related to the local agglomeration of excessive hard phases during solidification. These agglomerated regions weakened the structural continuity of the coating and acted as potential sites for strain mismatch and stress concentration [39]. Under reciprocating shear stress, cracks could initiate near the agglomerated regions and then propagate toward the worn surface, resulting in local brittle spalling rather than uniform material removal. Consequently, the wear-track depth and profile irregularity increased with increasing TiN content.

**Figure 10:**
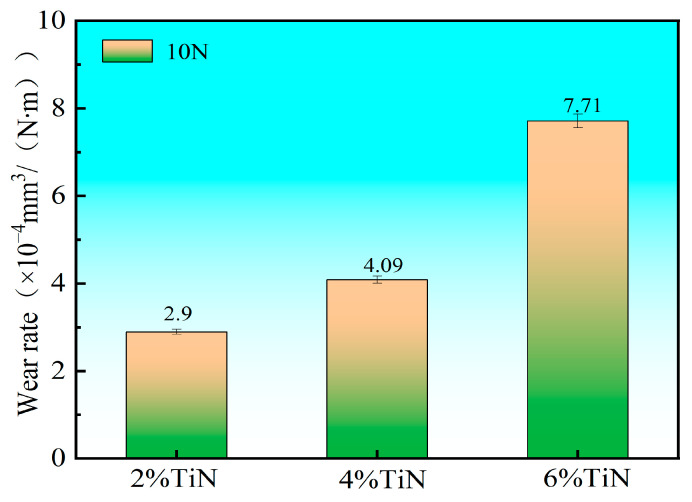
Comparison of wear rates of coatings for samples A, B, and C.

To further reveal the influence of TiN content on wear resistance, the wear rate of each coating under a 10 N load was calculated based on the wear volume, as summarized in Table 3 and shown in Figure 10. The wear volume was obtained from the cross-sectional profile method described in Section 2.4. The material loss below the unworn-surface reference line was integrated using Origin software, and the average cross-sectional area from three representative profiles was multiplied by the effective wear-track length. Comparing different compositions revealed that excessive addition of TiN actually weakened the wear resistance of the high-entropy alloy coating. This trend was consistent with the hardness test and surface morphology observations mentioned earlier. The results showed that at a 10 N load, the coating doped with 2% TiN exhibited the lowest wear rate, demonstrating the best wear resistance. An appropriate amount of TiN improved the load-bearing capacity and hardness of the coating mainly through dispersion strengthening and the relatively uniform distribution of Ti-containing phases. This helped reduce material loss during dry sliding and contributed to the lowest wear rate of the 2 wt.% TiN coating. This excellent interface bonding ensures that the coating maintains structural integrity and is less likely to undergo large-scale detachment when subjected to frictional wear. In contrast, a high proportion of TiN doping (4–6 wt.%) led to the coating entering a “brittle wear” mode, with the wear rate increasing sharply as the doping concentration increased. It can be seen that excessive TiN particles, due to the surface energy effect during the preparation process, are prone to agglomeration, which disrupts the continuity of the *AlCoCrFeNi* high-entropy alloy coating and severely reduces the material’s toughness. Under the friction load, the intense stress concentration at the edges of the agglomerated particles becomes the source of crack initiation, causing the coating to undergo non-continuous fragmentation and peeling before reaching the fatigue life. Thus, the addition of TiN has a significant “threshold effect” on wear resistance. Only when the distribution of the structure is uniform (such as 2 wt.%) can the hardness and toughness be optimally matched, thereby achieving the lowest wear rate.

**Figure 11:**
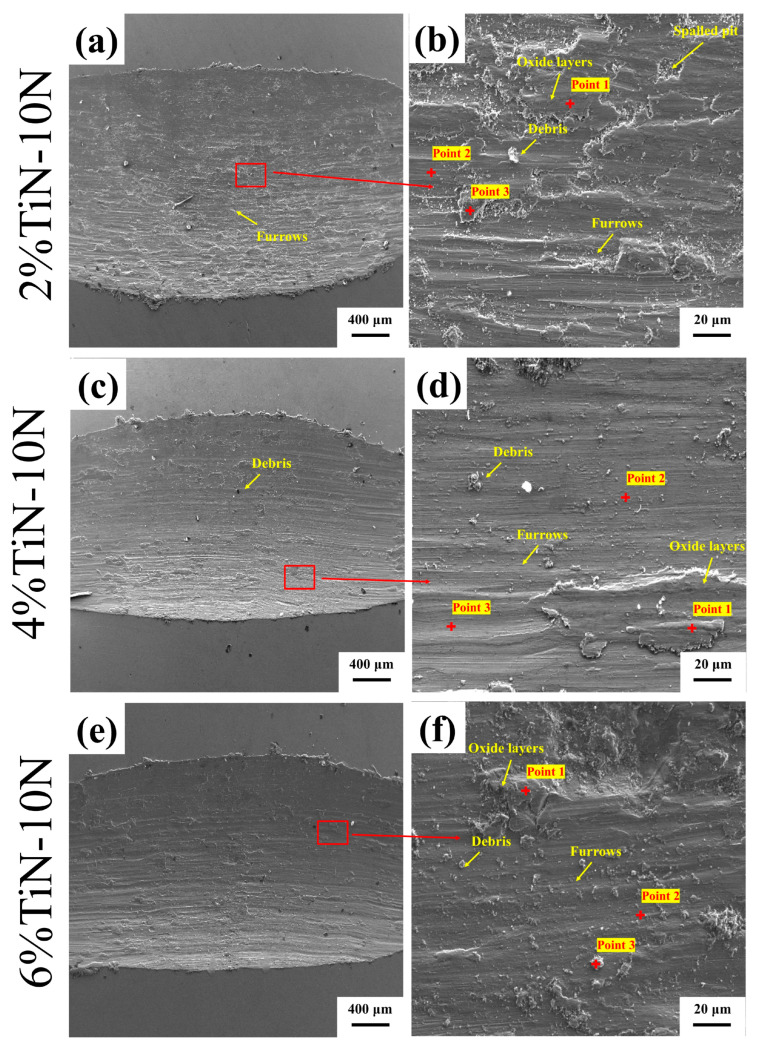
Worn-surface morphologies and EDS analysis locations of AlCoCrFeNi coatings with different TiN contents after dry sliding under a 10 N load: (**a**,**b**) 2 wt.% TiN; (**c**,**d**) 4 wt.% TiN; and (**e**,**f**) 6 wt.% TiN.

Through SEM microscopic morphology observation and EDS point analysis (Figure 11 and Table 4), the evolution law of the wear behavior of *AlCoCrFeNi* high-entropy alloy coatings with different TiN additions can be clearly revealed.

When the load is 10 N, the different TiN additions show significant differences in the ability to maintain the integrity of the coating surface. The worn-surface observations after the wear test (Figure 11a,b) indicate that the 2 wt.% TiN coating maintained a relatively flat surface, with the main damage features being ridges distributed along the sliding direction and scattered oxide debris. Combined with the data in the table, the oxygen content of the oxide coverage area on this surface remained at around 30.28%. The analysis suggests that under a 10 N load, the lower doping content of TiN provided sufficient supporting force by strengthening the matrix, enabling the material surface to form and maintain a continuous mechanical mixed layer, and the wear mechanism was mainly driven by oxidative wear [40,41,42].

As the TiN content further increases (4 wt.%), the damage mode of the coating begins to shift from a single oxidative wear to severe abrasive wear. Observing (Figure 11c,d), it is found that the surface grooves have significantly deepened and widened, and the oxide layer shows a tendency of local cracking and peeling. Although the oxygen content measured by EDS point analysis (Point 1) is as high as 42.37%, this is mainly due to the intensified wear that generates instantaneous friction heat, accelerating the oxidation of the metal. To discuss the reasons, the main factor is the higher proportion of the hard-phase TiN under a 10 N load, which triggers stress concentration, leading to an increase in the brittleness of the local oxide film. Under the action of periodic frictional forces, it is prone to breakage and peeling, and then turns into abrasive particles, inducing deeper damage.

When the doping amount of TiN reaches 6 wt.%, the combined effect of high load and high proportion of hard phase leads to a significant decline in the coating’s wear resistance, manifested by obvious fatigue spalling characteristics. As shown (Figure 11e,f), not only are there dense grooves on the surface but also large-scale spalled pits and delamination phenomena appear. Table 3 indicates that the oxygen content in these spalled areas and their surrounding regions (approximately 31.48–31.89%) has decreased compared to the 4 wt.% group. This reflects that, under a 10 N load, the interface between the excessive TiN particles and the substrate is subjected to shear stress impact, causing cracks to initiate and propagate beneath the surface, ultimately leading to the large-scale detachment of the oxide layer and the underlying substrate. At this point, the wear mechanism changes from oxidative wear to severe spalling wear.

In conclusion, under a 10 N load, an appropriate amount of TiN (2 wt.%) effectively inhibited plastic deformation and helped maintain a relatively stable oxide-containing mechanically mixed layer on the worn surface. In contrast, excessive TiN addition (4–6 wt.%) caused particle agglomeration and local stress concentration, which accelerated cracking and peeling of the surface oxide layer under repeated shear stress. The detached oxide fragments and hard particles then acted as abrasive debris, promoting three-body abrasive wear and brittle spalling. Therefore, the transition from a relatively retained oxide-containing region on the worn surface to discontinuous oxide-layer damage and hard-particle-induced abrasion is the main reason for the reduced wear resistance of coatings with higher TiN contents.

**Figure 12:**
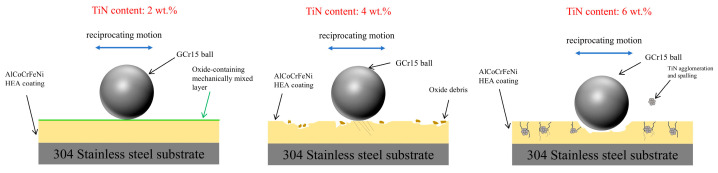
Schematic illustration of the wear mechanisms of AlCoCrFeNi coatings with different TiN contents.

The wear mechanism of the TiN-doped *AlCoCrFeNi* coatings is schematically illustrated in Figure 12. Based on the above experimental results, the wear behavior can be understood as a content-dependent transition from dispersion strengthening to agglomeration-induced damage. For the 2 wt.% TiN coating, the XRD results indicated a BCC-based phase structure, while the SEM/EDS observations showed a relatively uniform distribution of Ti-containing phases. This microstructural feature contributed to the effective dispersion-strengthening effect and improved load-bearing capacity of the coating, which was consistent with its highest hardness of 579.6 HV0.5. As a result, the 2 wt.% TiN coating exhibited the lowest and most stable friction coefficient, the shallowest wear track in the three-dimensional morphology, and the lowest wear rate. The worn-surface SEM/EDS results further indicated that the dominant wear mechanism was mainly mild oxidative wear accompanied by slight plastic deformation.

When the TiN content increased to 4 wt.%, the SEM/EDS results showed more obvious local enrichment of Ti-containing phases, and the worn surface exhibited deeper grooves and more debris. Although the hard phase could still provide a certain strengthening effect, the local aggregation reduced the structural uniformity of the coating and promoted stress concentration during reciprocating sliding. Therefore, the friction coefficient became less stable, the wear-track profile became deeper and wider, and the wear rate increased compared with the 2 wt.% TiN coating.

For the 6 wt.% TiN coating, excessive TiN addition led to more pronounced particle aggregation and microstructural discontinuity. These agglomerated regions acted as preferential sites for crack initiation under repeated shear stress. The cracks then propagated toward the worn surface, causing brittle spalling and the formation of hard debris. The detached particles participated in three-body abrasive wear between the GCr15 ball and the coating surface, resulting in severe friction fluctuations, obvious peeling pits, deeper wear tracks, and a higher wear rate. Therefore, the deterioration of wear resistance at high TiN contents can be attributed to the transition from dispersion strengthening to particle-agglomeration-induced brittle spalling and three-body abrasive wear.

## 4. Conclusions

1. TiN-doped *AlCoCrFeNi* high-entropy alloy coatings with different TiN contents were fabricated on 304 stainless steel by laser cladding. The coatings showed good metallurgical bonding with the substrate and were mainly composed of BCC-structured phases. With increasing TiN content, the BCC2-related diffraction peaks became more pronounced, and local Ti-rich aggregation was observed in the coating with higher TiN addition.
2. Within the investigated TiN-content range, the 2 wt.% TiN coating exhibited the best tribological performance under dry reciprocating sliding conditions at 10 N, 5 Hz, a stroke length of 5 mm, and a duration of 20 min. This coating showed the highest average hardness of 579.6 HV_0.5_, a relatively low and stable friction coefficient of approximately 0.30–0.35, and the lowest wear rate of 2.9 × 10^−4^ mm^3^/(N·m).
3. Excessive TiN addition reduced the wear resistance of the coatings. Compared with the 2 wt.% TiN coating, the 4 wt.% and 6 wt.% TiN coatings showed more severe friction fluctuations, deeper wear tracks, and more obvious peeling pits. The dominant wear mechanism gradually changed from mild oxidative wear and plastic deformation to brittle spalling and three-body abrasive wear.
4. This study focused on the effect of three TiN contents under a fixed laser cladding process and a single dry sliding condition. Therefore, the present results identify the best-performing coating only within the investigated TiN-content range. Future work will further examine intermediate TiN additions, different laser cladding parameters, and multiple wear-test conditions, such as different loads, sliding speeds, and environmental conditions, to further optimize the *AlCoCrFeNi-TiN* coating system.

## Figures and Tables

**Table 1:** Laser cladding parameter settings.

Parameters	Value
Laser power	1800 W
Scanning speed	6 mm/s
Powder feeding rate	10 g/min
Shielding gas flow rate	15 L/min
Spot diameter	4 mm

**Table 2:** SEM-EDS area analysis results of selected regions in the mixed powders with different TiN additions (at.%).

Sample	Elements (at.%)						
	Al	Co	Cr	Fe	Ni	Ti	N
AlCoCrFeNi-2%TiN	9.55	9.16	10.00	9.61	8.90	15.37	37.42
AlCoCrFeNi-4%TiN	7.20	7.65	8.59	8.16	7.36	22.82	38.22
AlCoCrFeNi-6%TiN	6.93	7.44	8.33	7.99	7.19	23.56	38.56

**Table 3:** Wear-track geometrical parameters and wear rates of the coatings under a 10 N load.

Sample	Maximum Wear Depth (μm)	Wear-Track Width (μm)	Wear Volume (mm^3^)	Wear Rate (mm^3^/(N·m))
2 wt.% TiN	60	1064	0.174	2.90 × 10^−4^
4 wt.% TiN	125	1482	0.245	4.09 × 10^−4^
6 wt.% TiN	159	1613	0.463	7.71 × 10^−4^

**Table 4:** EDS point analysis results (in at.%) of the high-entropy alloy coatings doped with three different contents of TiN after friction and wear under a 10 N load.

Sample	Region	Elements (at.%)							
		Al	Co	Cr	Fe	Ni	Ti	O	Si
AlCoCrFeNi-2%TiN	Point 1	7.97	7.77	13.26	29.96	9.90	0.50	30.28	0.35
	Point 2	10.61	11.18	18.74	42.52	13.96	2.46	-	0.52
	Point 3	10.83	11.29	18.72	43.39	14.74	0.64	-	0.38
AlCoCrFeNi-4%TiN	Point 1	6.04	5.59	10.70	27.03	7.52	0.42	42.37	0.33
	Point 2	8.67	8.84	18.74	49.96	12.90	0.38	-	0.52
	Point 3	8.33	8.03	14.41	34.30	10.65	0.48	23.28	0.51
AlCoCrFeNi-6%TiN	Point 1	5.37	5.73	12.54	34.65	8.42	1.43	31.48	0.37
	Point 2	6.94	8.11	18.33	53.57	12.14	0.34	-	0.56
	Point 3	5.39	5.52	12.72	35.43	8.06	0.35	31.89	0.64

## Data Availability

The original contributions presented in this study are included in the article. Further inquiries can be directed to the corresponding authors.
